# Crystal structure of 4-amino-2,6-di­chloro­phenol

**DOI:** 10.1107/S2056989015009172

**Published:** 2015-05-20

**Authors:** Kyle J. McDonald, Vasumathi Desikan, James A. Golen, David R. Manke

**Affiliations:** a Department of Science & Math, Massasoit Community College, 1 Massasoit Boulevard, Brockton, MA 02302, USA; b Department of Chemistry and Biochemistry, University of Massachusetts Dartmouth, 285 Old Westport Road, North Dartmouth, MA 02747, USA

**Keywords:** crystal structure, amino­phenols, hydrogen bonding

## Abstract

The title compound, C_6_H_5_Cl_2_NO, has a single planar mol­ecule in the asymmetric unit with the non-H atoms possessing a mean deviation from planarity of 0.020 Å. In the crystal, O—H⋯N hydrogen bonds lead to the formation of infinite chains along [101] which are further linked by N—H⋯O hydrogen bonds, forming (010) sheets.

## Related literature   

For the crystal structure of the parent *p*-amino­phenol, see: Brown (1951 ▸). For other related structures, see: Ermer & Eling (1994 ▸); Dey *et al.* (2005 ▸); Bacchi *et al.* (2009 ▸).
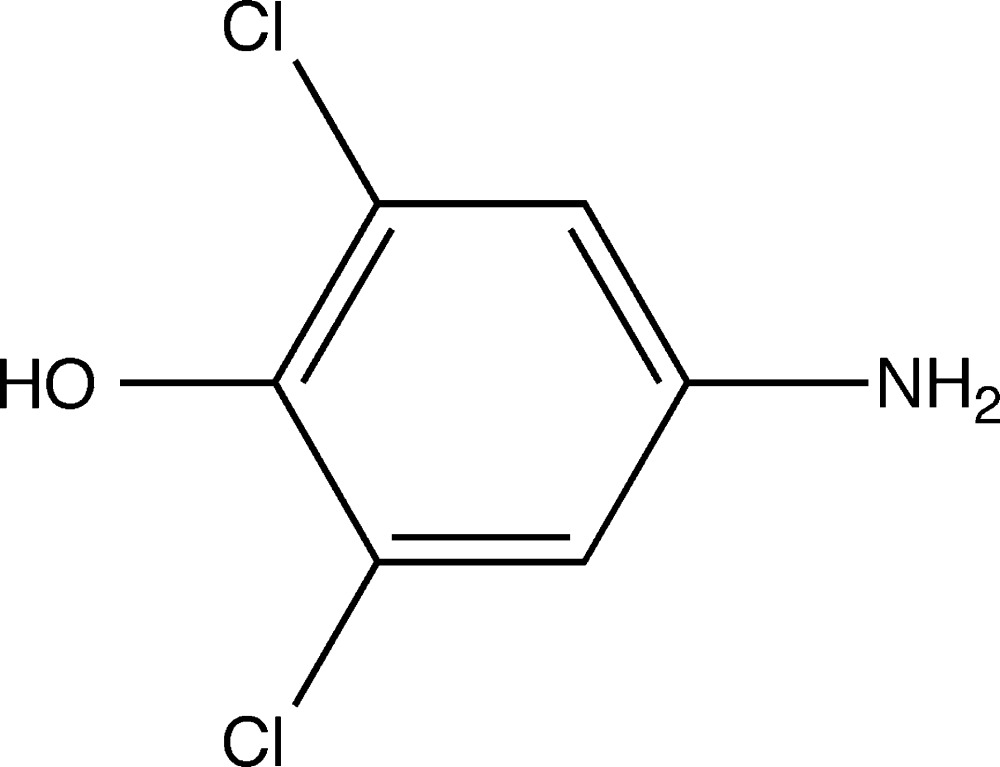


## Experimental   

### Crystal data  

C_6_H_5_Cl_2_NO
*M* *_r_* = 178.02
Monoclinic, 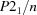

*a* = 4.6064 (5) Å
*b* = 11.7569 (12) Å
*c* = 13.2291 (13) Å
β = 96.760 (5)°
*V* = 711.47 (13) Å^3^
*Z* = 4
Cu *K*α radiation
μ = 7.59 mm^−1^
*T* = 120 K
0.4 × 0.2 × 0.1 mm

### Data collection  

Bruker D8 Venture CMOS diffractometer
Absorption correction: multi-scan (*SADABS*; Bruker, 2014 ▸) *T*_min_ = 0.425, *T*_max_ = 0.754
7481 measured reflections
1402 independent reflections
1273 reflections with *I* ≥ 2σ(*I*)
*R*_int_ = 0.043

### Refinement  

*R*[*F* ^2^ > 2σ(*F* ^2^)] = 0.033
*wR*(*F* ^2^) = 0.091
*S* = 1.05
1402 reflections
99 parameters
2 restraints
H atoms treated by a mixture of independent and constrained refinement
Δρ_max_ = 0.33 e Å^−3^
Δρ_min_ = −0.35 e Å^−3^

### 

Data collection: *APEX2* (Bruker, 2014 ▸); cell refinement: *SAINT* (Bruker, 2014 ▸); data reduction: *SAINT*; program(s) used to solve structure: *SHELXS97* (Sheldrick, 2008 ▸); program(s) used to refine structure: *SHELXL2014* (Sheldrick, 2015 ▸) and *OLEX2.refine* (Bourhis *et al.*, 2015 ▸); molecular graphics: *OLEX2* (Dolomanov *et al.*, 2009 ▸); software used to prepare material for publication: *OLEX2* and *publCIF* (Westrip, 2010 ▸).

## Supplementary Material

Crystal structure: contains datablock(s) I. DOI: 10.1107/S2056989015009172/ff2137sup1.cif

Structure factors: contains datablock(s) I. DOI: 10.1107/S2056989015009172/ff2137Isup2.hkl

Supporting information file. DOI: 10.1107/S2056989015009172/ff2137Isup3.cml

**Fig. 1.**
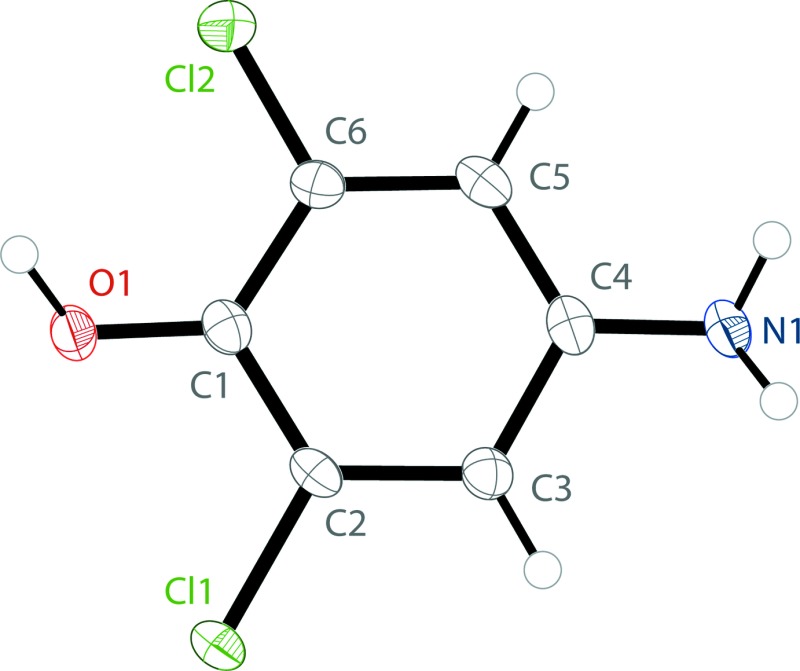
Molecular structure of the title compound, showing the atom-labelling scheme. Displacement ellipsoids are drawn at the 50% probability level. H atoms are drawn as spheres of arbitrary radius.

. DOI: 10.1107/S2056989015009172/ff2137fig1.tif
Mol­ecular structure of the title compound, showing the atom-labelling scheme. Displacement ellipsoids are drawn at the 50% probability level. H atoms are drawn as spheres of arbitrary radius.

**Fig. 2.**
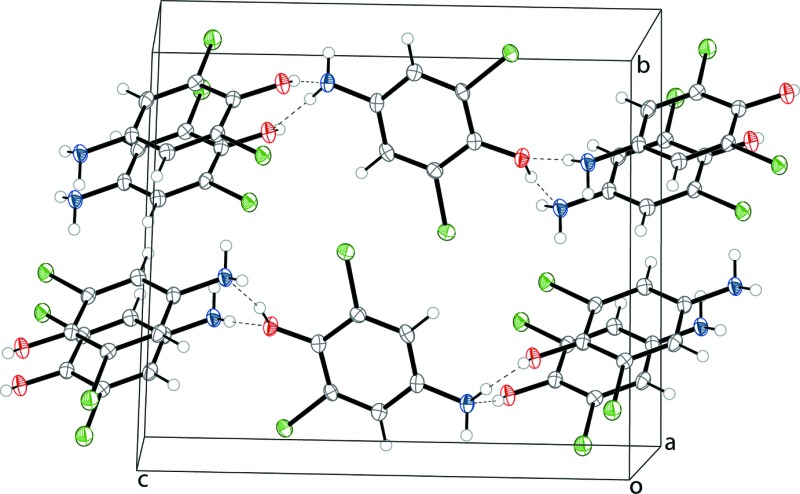
Molecular packing of the title compound with hydrogen bonding shown as dashed lines.

. DOI: 10.1107/S2056989015009172/ff2137fig2.tif
Mol­ecular packing of the title compound with hydrogen bonding shown as dashed lines.

CCDC reference: 1400729

Additional supporting information:  crystallographic information; 3D view; checkCIF report

## Figures and Tables

**Table 1:** Hydrogen-bond geometry (, )

*D*H*A*	*D*H	H*A*	*D* *A*	*D*H*A*
O1H1N1^i^	0.85(2)	1.82(2)	2.653(2)	168(2)
N1H1*a*O1^ii^	0.87(1)	2.05(1)	2.921(2)	177(2)

Symmetry codes: (i) 
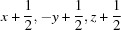
; (ii) 
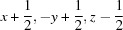
.

## supplementary crystallographic information

### S1. Comment

The hydrogen bonding networks of amino­phenols have been explored as hy­droxy and amino groups are complementary hydrogen bonding donors and acceptors. This is exemplified in *p*-amino­phenol, which exhibits a supertetra­hedral hydrogen bonded architecture where all hydrogen bonding donors and acceptors are saturated (Brown, 1951; Ermer *et al.*, 1994). The mono-substitution in 4-amino-2-methyl­phenol and 4-amino-3-methyl­phenol yields a square motif structure that again exhibits saturation among hydrogen bonding donors and acceptors (Dey *et al.*, 2005). The more sterically encumbered substitution of 4-amino-2,6-di­phenyl­phenol prevents the saturation in hydrogen bonding, with only O–H···N and N–H···aryl inter­actions observed (Bacchi *et al.*, 2009). The 2,6-di­chloro substitution of the title compound also prevents saturation in its hydrogen bonding network.

The molecular structure of the title compound demonstrates a planar molecule with a mean deviation from the plane of the non-hydrogen atoms of 0.020 Å. Inter­molecular hydrogen bonding between O1–H1···N1 results in infinite chains along [101] which combine with inter­molecular hydrogen bonding between N1–H1a···O1 to give (010) sheets. The packing for the title compound indicating hydrogen bonding is shown in Figure 2.

### S2. Experimental

A commercial sample (Aldrich) was used for the crystallization. Crystals suitable for single crystal X-ray analysis were grown by slow evaporation of a methanol solution.

### S3. Refinement

All non-hydrogen atoms were refined anisotropically (Olex2) by full matrix least squares on F^2^. Hydrogen atoms H1, H1a and H1b were found from a Fourier difference map. H1 was allowed to refine freely with an isotropic displacement parameter of 1.20 times *U*_eq_ of the parent O atom. H1a and H1b were refined with a fixed distance of 0.87 (0.005) Å and isotropic displacement parameters of 1.20 times *U*_eq_ of the parent N atom. The two remaining hydrogen atoms were placed in calculated positions and then refined with riding model with C–H lengths of 0.95 Å with isotropic displacement parameters set to 1.20 times *U*_eq_ of the parent C atom.

### Crystal data

**Table:**

C_6_H_5_Cl_2_NO	*F*(000) = 363.7579
*M**_r_* = 178.02	*D*_x_ = 1.662 Mg m^−^^3^
Monoclinic, *P*2_1_/*n*	Cu *K*α radiation, λ = 1.54178 Å
Hall symbol: -P 2yn	Cell parameters from 5198 reflections
*a* = 4.6064 (5) Å	θ = 5.1–72.2°
*b* = 11.7569 (12) Å	µ = 7.59 mm^−^^1^
*c* = 13.2291 (13) Å	*T* = 120 K
β = 96.760 (5)°	Plate, colourless
*V* = 711.47 (13) Å^3^	0.4 × 0.2 × 0.1 mm
*Z* = 4	

### Data collection

**Table:**

Bruker D8 Venture CMOS diffractometer	1402 independent reflections
Radiation source: microfocus Cu	1273 reflections with *I*≥ 2σ(*I*)
HELIOS MX monochromator	*R*_int_ = 0.043
Detector resolution: 102.4 pixels mm^-1^	θ_max_ = 72.2°, θ_min_ = 5.1°
φ and ω scans	*h* = −5→5
Absorption correction: multi-scan (*SADABS*; Bruker, 2014)	*k* = −14→14
*T*_min_ = 0.425, *T*_max_ = 0.754	*l* = −12→16
7481 measured reflections	

### Refinement

**Table:**

Refinement on *F*^2^	2 restraints
Least-squares matrix: full	7 constraints
*R*[*F*^2^ > 2σ(*F*^2^)] = 0.033	H atoms treated by a mixture of independent and constrained refinement
*wR*(*F*^2^) = 0.091	*w* = 1/[σ^2^(*F*_o_^2^) + (0.0618*P*)^2^ + 0.1912*P*] where *P* = (*F*_o_^2^ + 2*F*_c_^2^)/3
*S* = 1.05	(Δ/σ)_max_ < 0.001
1402 reflections	Δρ_max_ = 0.33 e Å^−^^3^
99 parameters	Δρ_min_ = −0.35 e Å^−^^3^

### Special details

**Table:**

Experimental. Absorption correction: SADABS-2014/4 (Bruker, 2014) was used for absorption correction. wR2(int) was 0.1370 before and 0.0641 after correction. The Ratio of minimum to maximum transmission is 0.5642. The λ/2 correction factor is 0.00150.

### Fractional atomic coordinates and isotropic or equivalent isotropic displacement parameters (Å^2^)

**Table:**

	*x*	*y*	*z*	*U*_iso_*/*U*_eq_	
Cl1	0.10618 (10)	0.09750 (4)	0.70434 (3)	0.02213 (17)	
Cl2	0.79187 (11)	0.44532 (4)	0.61176 (4)	0.02666 (18)	
O1	0.4519 (3)	0.30303 (12)	0.74687 (10)	0.0214 (3)	
N1	0.4522 (4)	0.13356 (14)	0.35171 (12)	0.0199 (4)	
C1	0.4563 (4)	0.26523 (16)	0.65055 (14)	0.0176 (4)	
C4	0.4607 (4)	0.17796 (16)	0.45234 (13)	0.0176 (4)	
C5	0.6112 (4)	0.27756 (16)	0.48060 (14)	0.0196 (4)	
H5	0.7170 (4)	0.31620 (16)	0.43359 (14)	0.0235 (5)*	
C2	0.3011 (4)	0.16753 (16)	0.61822 (14)	0.0171 (4)	
C3	0.3014 (4)	0.12337 (16)	0.52110 (14)	0.0182 (4)	
H3	0.1938 (4)	0.05638 (16)	0.50165 (14)	0.0219 (5)*	
C6	0.6052 (4)	0.31990 (16)	0.57788 (14)	0.0179 (4)	
H1	0.615 (5)	0.329 (2)	0.7732 (18)	0.0215 (5)*	
H1a	0.601 (3)	0.1551 (19)	0.3217 (15)	0.0215 (5)*	
H1b	0.451 (5)	0.0598 (4)	0.3523 (17)	0.0215 (5)*	

### Atomic displacement parameters (Å^2^)

**Table:**

	*U*^11^	*U*^22^	*U*^33^	*U*^12^	*U*^13^	*U*^23^
Cl1	0.0232 (3)	0.0246 (3)	0.0204 (3)	−0.00409 (16)	0.00988 (19)	0.00083 (17)
Cl2	0.0334 (3)	0.0245 (3)	0.0225 (3)	−0.01014 (19)	0.0052 (2)	−0.00026 (18)
O1	0.0193 (6)	0.0298 (7)	0.0156 (7)	−0.0039 (6)	0.0046 (5)	−0.0042 (6)
N1	0.0206 (8)	0.0247 (9)	0.0151 (8)	0.0013 (6)	0.0057 (6)	−0.0009 (6)
C1	0.0146 (8)	0.0225 (9)	0.0159 (9)	0.0027 (7)	0.0023 (7)	0.0008 (7)
C4	0.0138 (8)	0.0248 (9)	0.0142 (9)	0.0050 (7)	0.0009 (7)	0.0005 (7)
C5	0.0177 (9)	0.0244 (10)	0.0173 (9)	0.0005 (7)	0.0048 (7)	0.0054 (7)
C2	0.0144 (8)	0.0215 (9)	0.0163 (9)	0.0011 (7)	0.0051 (7)	0.0035 (7)
C3	0.0148 (8)	0.0217 (9)	0.0185 (10)	0.0005 (7)	0.0029 (7)	−0.0007 (7)
C6	0.0158 (8)	0.0188 (9)	0.0194 (9)	−0.0007 (7)	0.0026 (7)	0.0016 (7)

### Geometric parameters (Å, º)

**Table:**

Cl1—C2	1.7387 (18)	C1—C6	1.401 (3)
Cl2—C6	1.7389 (19)	C4—C5	1.389 (3)
O1—C1	1.352 (2)	C4—C3	1.391 (3)
O1—H1	0.85 (2)	C5—H5	0.9500
N1—C4	1.426 (2)	C5—C6	1.383 (3)
N1—H1a	0.870 (5)	C2—C3	1.386 (3)
N1—H1b	0.867 (5)	C3—H3	0.9500
C1—C2	1.393 (3)		
			
H1—O1—C1	113.3 (16)	C6—C5—C4	119.33 (17)
H1a—N1—C4	112.6 (15)	C6—C5—H5	120.34 (11)
H1b—N1—C4	110.9 (15)	C1—C2—Cl1	118.37 (14)
H1b—N1—H1a	108 (2)	C3—C2—Cl1	119.15 (14)
C2—C1—O1	119.75 (16)	C3—C2—C1	122.47 (16)
C6—C1—O1	123.97 (17)	C2—C3—C4	119.44 (18)
C6—C1—C2	116.26 (17)	H3—C3—C4	120.28 (11)
C5—C4—N1	121.18 (17)	H3—C3—C2	120.28 (11)
C3—C4—N1	118.87 (18)	C1—C6—Cl2	118.64 (14)
C3—C4—C5	119.85 (17)	C5—C6—Cl2	118.79 (14)
H5—C5—C4	120.34 (10)	C5—C6—C1	122.57 (17)
			
Cl1—C2—C3—C4	−178.98 (14)	C4—C5—C6—Cl2	−179.43 (14)
O1—C1—C2—Cl1	−0.1 (2)	C4—C5—C6—C1	1.0 (3)
O1—C1—C2—C3	−178.74 (17)	C5—C4—C3—C2	−1.7 (3)
O1—C1—C6—Cl2	−1.1 (3)	C2—C1—C6—Cl2	177.57 (13)
O1—C1—C6—C5	178.43 (17)	C2—C1—C6—C5	−2.9 (3)
N1—C4—C5—C6	177.70 (17)	C3—C4—C5—C6	1.3 (3)
N1—C4—C3—C2	−178.14 (17)	C6—C1—C2—Cl1	−178.80 (13)
C1—C2—C3—C4	−0.3 (3)	C6—C1—C2—C3	2.5 (3)

### Hydrogen-bond geometry (Å, º)

**Table:**

*D*—H···*A*	*D*—H	H···*A*	*D*···*A*	*D*—H···*A*
O1—H1···N1^i^	0.85 (2)	1.82 (2)	2.653 (2)	168 (2)
N1—H1*a*···O1^ii^	0.87 (1)	2.05 (1)	2.921 (2)	177 (2)

Symmetry codes: (i) *x*+1/2, −*y*+1/2, *z*+1/2; (ii) *x*+1/2, −*y*+1/2, *z*−1/2.
