# Supplementary figures and images for: Crystal structure of 4-amino-2,6-di­chloro­phenol
Source: Acta Crystallogr E Crystallogr Commun. 2015 May 20;71(Pt 6):o406. doi: 10.1107/S2056989015009172 (PMC4459336; doi:10.1107/S2056989015009172)

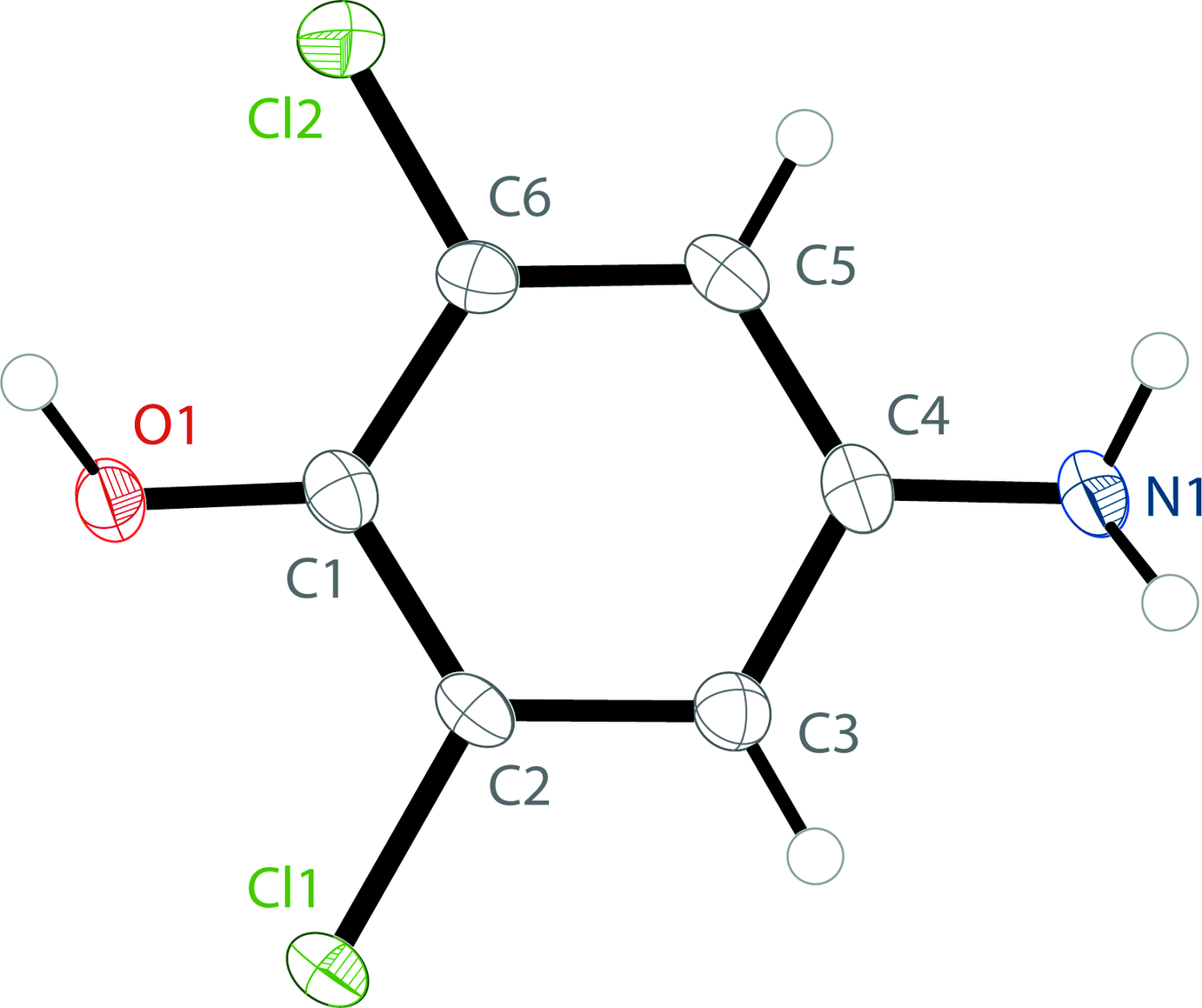

Supplement: Supplementary file 4 [file e-71-0o406-fig1.tif]

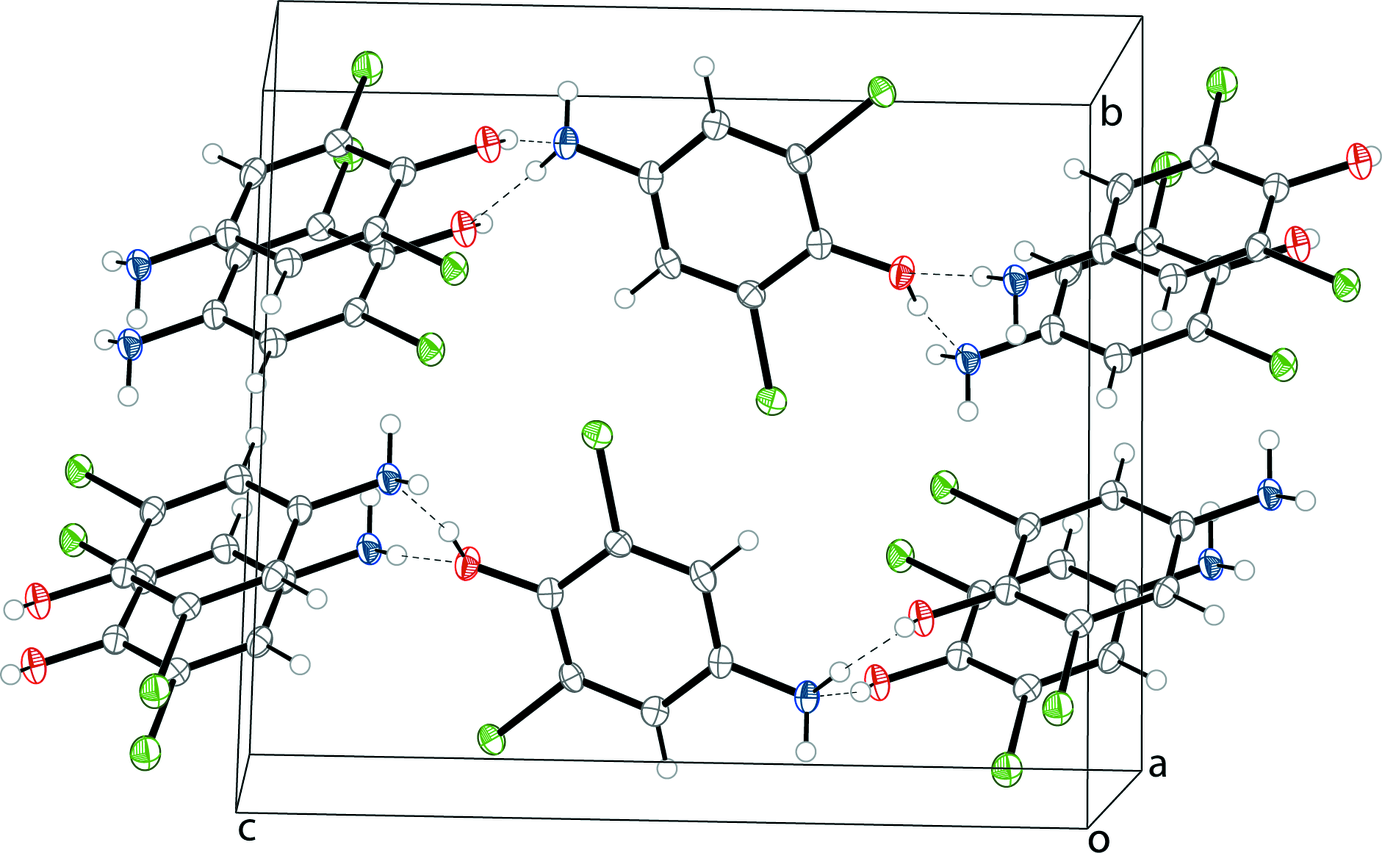

Supplement: Supplementary file 5 [file e-71-0o406-fig2.tif]
